# Grey matter abnormalities in Tourette syndrome: an activation likelihood estimation meta-analysis

**DOI:** 10.1186/s12888-021-03187-1

**Published:** 2021-04-07

**Authors:** Fang Wen, Junjuan Yan, Liping Yu, Fang Wang, Jingran Liu, Ying Li, Yonghua Cui

**Affiliations:** Department of Psychiatry, Beijing Children’s Hospital, Capital Medical University, National Center for Children Healthy, 56 Nanlishi Road, Beijing, China

**Keywords:** Grey matter, Tourette syndrome, ALE, Voxel-based morphometry, Thalamus

## Abstract

**Background:**

Tourette syndrome (TS) is a neurodevelopmental disorder defined by the continual presence of primary motor and vocal tics. Grey matter abnormalities have been identified in numerous studies of TS, but conflicting results have been reported. This study was an unbiased statistical meta-analysis of published neuroimaging studies of TS structures.

**Methods:**

A voxel quantitative meta-analysis technique called activation likelihood estimation (ALE) was used. The meta-analysis included six neuroimaging studies involving 247 TS patients and 236 healthy controls. A statistical threshold of *p* < 0.05 was established based on the false discovery rate and a cluster extent threshold of 50 voxels.

**Results:**

We found that grey matter volumes were significantly increased in the bilateral thalamus, right hypothalamus, right precentral gyrus, left postcentral gyrus, left inferior parietal lobule, right lentiform nucleus, and left insula of TS patients compared to those of healthy controls. In contrast, grey matter volumes were significantly decreased in the bilateral postcentral gyrus, bilateral anterior cingulate, bilateral insula, left posterior cingulate and left postcentral gyrus of TS patients compared to those of healthy controls.

**Conclusions:**

Our present meta-analysis primarily revealed significant increases in grey matter volumes in the thalamus and lentiform nucleus, and decreased grey matter volumes in the anterior cingulate gyrus, of TS patients compared to those in healthy controls. Most of these identified regions are associated with cortico-striato-thalamo-cortical circuits. Further studies with larger sample sizes are needed to confirm these changes in grey matter volumes in TS patients.

**Supplementary Information:**

The online version contains supplementary material available at 10.1186/s12888-021-03187-1.

## Background

Tourette syndrome (TS) is a neurodevelopmental disorder defined by the continual presence of primary motor and vocal tics [1]. TS is mainly diagnosed by observing symptoms and by referring to disease history [2]. Magnetic resonance imaging (MRI) has enabled early diagnosis, treatment, evaluation, and pathogenic studies of TS. It has been suggested that TS arises from neurobiological abnormalities, of which imaging studies have revealed many relevant clues [3, 4]. For example, basal ganglia grey matter volume decreased and dorsolateral prefrontal area increased in children with TS [5]. Smaller corpus-callosum volumes and thinner sensorimotor cortices have been also reported in structural imaging studies on TS [6, 7]. It indicated that some of these regions with grey matter abnormalities may be associated with cortico-striato-thalamo-cortical (CSTC) circuits in TS [5, 8, 9]. However, identification of the specific regions of grey matter abnormalities linked to the CSTC circuit of TS requires further investigation.

Moreover, previous imaging studies on grey matter abnormalities in TS patients have yielded conflicting results. For instance, in previous imaging studies in TS patients, grey matter volumes are decreased in the basal ganglia and left hippocampal gyrus, as well as in orbitofrontal, anterior cingulate, and bilateral ventrolateral prefrontal cortices [10], whereas such volumes are increased in dorsal-lateral prefrontal regions [10, 11], the bilateral ventral putamen [12], posterior thalamus, and hypothalamus. However, decreased grey matter volumes have also been reported in the putamen and some prefrontal regions [13]. Hence, these conflicting findings indicate that grey matter abnormalities in TS patients remain unclear and that further studies are needed.

Therefore, in the present study, we performed a meta-analysis on the results of studies reporting grey matter abnormalities in TS patients. Activation likelihood estimation (ALE), a quantitative meta-analysis for imaging studies, is a statistically-based approach that we used to analyze results of grey matter volumes across studies. Due to most published studies on TS using voxel-based morphometry (VBM) to assess grey matter volumes, we performed VBM-based ALE analysis to assess previous results of grey matter volumes in TS patients.

## Methods

### Study selection

PubMed, Web of Knowledge, Elsevier, and PsycINFO were searched for articles published between January 31, 2006, and December 31, 2019. The following keywords were used: Tourette syndrome, MRI; Tic disorder, MRI; TS, MRI; Tourette syndrome, grey matter; Tic disorder, grey matter; TS, grey matter. The inclusion criteria for articles were as follows: (1) TS diagnosis based on either the Diagnostic and Statistical Manual of Mental Disorders, 4th Edition, text revision (DSM-IV-TR) or DSM-5 criteria for TS; (2) Grey matter changes were reported by volumes, thicknesses, or densities in structural MRIs; (3) The foci were reported in either Talairach space or the Montreal Neurological Institute (MNI) space; (4) The search was confined to English- language articles. The exclusion criteria for articles were as follows: (1) duplicated studies; (2) articles that were not original studies; or (3) no coordinates of grey matter were reported.

**Fig. 1:**
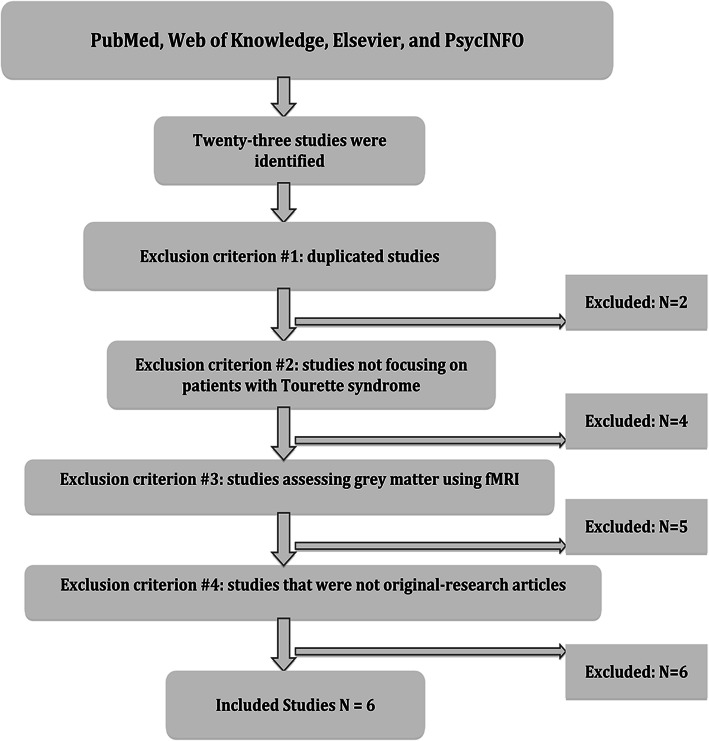
The flowchart for the identification of the included study

Ultimately, a total of six studies (247 TS patients and 236 healthy controls) were included in the present meta-analysis. Figure 1 shows a flowchart depicting our steps in the identification of relevant studies. Information on ages, percentages of males, coordinates, measures, Yale Global Tic Severity Scale (YGTSS) values, and the main findings of each study were extracted (Table 1). Additionally, a total of 6 fMRI studies of tic disorders were identified. The main findings of each fMRI were present in sTable 1(Supplementary Material).

**Table 1:** Demographic characteristics of subjects and Summary of articles included in ALE meta-analysis of TS

First Author	Year	YGTSS ($\overline{x}$ ±s)	N	Age($\overline{x}$ ±s)	Males (%)	DOI	TS, HC	Coordinates	Measure	Main Findings
Greene	2017	18.1 ± 8.3	103	11.9 ± 2.1	78.64	N/A	103, 103	MNI	GMV	The TS group demonstrated lower WM volumes bilaterally in orbital and medial prefrontal cortices, and greater GM volumes in the posterior thalamus, hypothalamus, and midbrain. In a multiple regression analysis for the relative volume in each significant cluster, we modeled YGTSS and age as factors and their interaction. The YGTSS effect and the YGTSS × age interaction was not significant in any of the models, suggesting our results reflected diagnosis rather than cross-sectional tic severity.
Muellner	2015	17.64 ± 7.05	52	29.5 ± 8.51	55.77	N/A	52, 52	MNI	N/A	Lower depth and reduced thickness of gray matter was found in the pre- and post-central gyri, as well as superior, inferior, and internal frontal sulci. A negative correlation of YGTSS/50 score with sulcal depth of the superior temporal sulcus and central sulcus on the right.
Draper	2015	N/A	35	14 ± 3.1	91.43	N/A	35, 29	MNI	GMT	PSP was inversely associated with grey matter thickness within insular and sensorimotor cortices. Grey matter thickness was significantly reduced in these areas in individuals with TS. PSP ratings were significantly correlated with tic severity. Premonitory Urge for Tics Scale scores that were negatively correlated with grey matter thickness were identified in clusters located within sensorimotor cortical areas and within the left insular cortex. By contrast, there were no significant clusters of positive correlations between PUTS scores and cortical grey matter thickness.
Liu	2013	41.71 ± 12.46	21	7.9 ± 1.95	95.24	1.84 ± 0.56	21, 20	MNI	GMV	Brain volume changes were found in the left superior temporal gyrus, bilateral paracentral gyrus, right precuneous cortex, right pre- and post-central gyrus, left temporal-occipital fusiform cortex, right frontal pole, and left lingual gyrus in TS patients. Increases were found in the anterior thalamic radiation, right cingulum bundle projecting to the cingulate gurus, and the forceps minor. Decreases in white matter volume (WMV) were found in the right frontal pole. No correlations between gray matter volume change and YGTSS scores or tic duration were found.
Draganski	2010	28.7 ± 7.4	40	32.4 ± 11	75	24 ± 11.6	40,40	MNI	GMV& GMT	Reductions in grey matter volume were found in orbitofrontal, anterior cingulate, and ventrolateral prefrontal cortices bilaterally. Cortical thinning extended into the limbic mesial temporal lobe. The VBM analysis failed to demonstrate any significant correlation between grey matter volume and overall tic severity (YGTSS).
Müller-Vahl	2009	28.8	19	30.4	100	N/A	19, 20	MNI	GMV	Decreases in gray matter volumes were found in prefrontal areas, the anterior cingulate gyrus, sensorimotor areas, left caudate nucleus, and left postcentral gyrus. Decreases in white matter volumes were detected in the right inferior frontal gyrus, left superior frontal gyrus, and the anterior corpus callosum. Increases were found in the left middle frontal gyrus and left sensorimotor areas. In MTI, white matter reductions were seen in the right medial frontal gyrus and inferior frontal gyrus bilaterally, as well as in the right cingulate gyrus. The YGTSS score was negatively correlated with GMV.

Abbreviations: YGTSS, Yale Global Tic Severity Scale; DOI, Duration Of Illness; HC, Health Control; MNI, Montreal Neurological Institute; GMV, Grey Matter Volume; WMV, White Matter Volume; GMT, Grey Matter Thickness; PSP, Premonitory Sensory Phenomena

### Activation likelihood estimation (ALE)

Neuroimaging studies usually report the peak coordinates (FOCI) of the activated brain regions under several contrast conditions. Finding the consistency of activation positions in multiple studies is more in line with the characteristics of current neuroimaging research. Therefore, ale analysis based on the coordinate data reported in the literature has gradually become the mainstream of data element analysis of neuroimaging [14].

ALE is to estimate the probability of co-activation of various brain regions in a certain task state. ALE method firstly calculates the activation possibility of each voxel in the whole brain under certain conditions in each experiment and then tests the stability of voxel activation across experiments [15]. ALE method assumes that the possibility that the whole brain voxels are included in the brain area represented by a certain activation peak point is assumed as follows: this possibility is centred on the activation peak point and presents a 3D Gaussian distribution [16]. In this distribution, voxels close to the activation peak are more likely to be activated than those far from the activation peak. Based on this idea, the ALE method can calculate the activation possibility of each voxel in the whole brain. All meta-analysis was accomplished by applying the ALE technique [17], which has been implemented in the Brain Map [18]. ALE analyses were carried out in Talairach space, and a Lancaster transform was conducted if coordinates were instead originally reported in Montreal Neurological Institute (MNI) space [19]. Brett’s formula [20] and the Lancaster method were used in the transition from MNI space to Talairach space. ALE analysis of TS included six studies comprised of 208 foci. The resulting statistical maps were corrected for multiple comparisons using false discovery rates (FDRs) and were then thresholded at *p* < 0.05, with a cluster-extent threshold of 50 voxels.

## Results

**Fig. 2:**
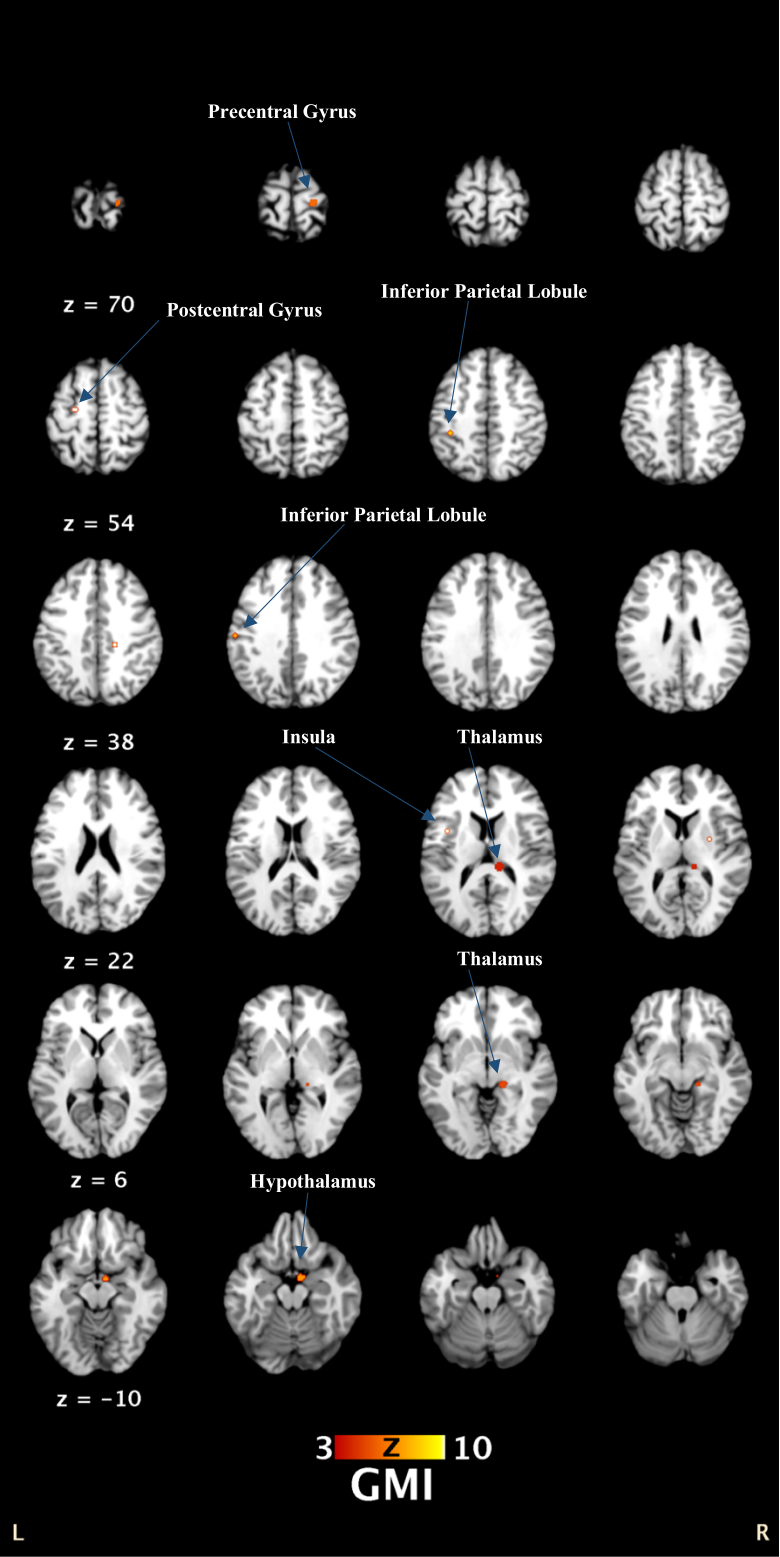
Results from the ALE meta-analysis on grey matter increased volumes in patients with TS compared with healthy controls

ALE results showed that grey matter volumes were significantly increased in the left thalamus, left red nucleus, right thalamus, right hypothalamus, right precentral gyrus, left postcentral gyrus, left inferior parietal lobule, right putamen, and left insula of TS patients compared to those of healthy controls. In contrast, grey matter volumes were significantly decreased in the bilateral postcentral gyrus, bilateral anterior cingulate, bilateral insula, left posterior cingulate and left postcentral gyrus of TS patients compared to those of healthy controls. For more details, see Table 2 and Fig. 2.

**Table 2:** Results of ALE analyses on grey matter abnormalities in TS patients

Cluster#	Volume (mm^**3**^)	Peak ALE value	Z values	Talairach coordinates(x, y, z)	Brain regions
**Groups with increased grey matter volumes**					
1	952	0.019318 0.016552817	5.344082	−12, −30, 10 −14, −28, − 2	(L) Thalamus (61.3% Pulvinar, 2.5% Medial Geniculate Body)
			4.7613444		
2	784	0.01593744 0.015419524	4.662963	−2, −6, −6 0, −16, −8	(L) Thalamus (Red Nucleus)
			4.5910673		
3	480	0.019446805	5.3822155	12, −30,13	(R) Thalamus (100% Pulvinar)
4	424	0.016458694	4.751242	16, −28, −2	(R) Thalamus (16.7% Pulvinar)
5	384	0.015193136	4.555133	20, −22, 68	(R) Precentral Gyrus (BA 4)
6	336	0.014851548	4.504367	8, −4, −14	(R) Hypothalamus
7	80	0.010117263	3.6241639	−56, −18, 33	(L) Postcentral Gyrus (BA 2)
8	80	0.010117263	3.6241639	−36, −34, 45	(L) Inferior Parietal Lobule (BA 40)
9	64	0.008642835	3.1104991	27, −3, 9	(R) Lentiform Nucleus (Putamen)
10	64	0.00938297	3.3538606	−38, 4, 12	(L) Insula (BA 13)
**Groups with decreased grey matter volumes**					
1	208	0.00997	3.9280179	−56, −18, 34	(L) Postcentral Gyrus (BA 2)
2	96	0.008955	3.6178029	6, 44, 0	(R) Anterior Cingulate (BA 32&24)
3	96	0.009334	3.8218374	−40, −2, 8	(L) Sub-lobar (Insula)
4	80	0.009279	3.7407575	0, 33, −26	(L) Medial Frontal Gyrus (BA 11)
5	80	0.008896	3.611275	−40, 4, 12	(L) Sub-lobar (Insula)
6	80	0.009279	3.7407575	58, −13, 16	(R) Postcentral Gyrus (BA 43)
7	56	0.009123	3.6994894	−14, 40, 10	(L) Anterior Cingulate (BA 32)
8	56	0.009123	3.6994894	−8, −52, 26	(L) Posterior Cingulate (BA 31)

Abbreviations: ALE, activation likelihood estimation; BA, Brodmann area; L, left; R, right

## Discussion

To our knowledge, this is the first ALE analysis of VBM-based MRI studies in TS patients. The main findings of the present study were that grey matter volumes in the thalamus, precentral gyrus, postcentral gyrus, and lentiform nucleus were increased, whereas such volumes were decreased in the anterior cingulate and medial frontal gyrus of TS patients.

### Thalamus

Several imaging studies have found increased total thalamic volumes in children and adults with TS [21]. In the present study, we found that grey matter volume was increased in the thalamus of TS patients, especially within the pulvinar nucleus. The pulvinar nucleus is the largest thalamic nucleus and has robust connectivity with the visual cortex [22]. Previous studies have shown that the most prominent differences in grey matter volumes between TS patients and healthy controls were in the pulvinar nucleus and several other motor nuclei [13]. There is a bidirectional connection between the pulvinar nucleus and the cortex [22]. The medial nucleus pulposus is extensively connected with the cortex, including the prefrontal cortex, orbital cortex and cingulate cortex [23]. Some regions that connect with the pulvinar nucleus are also associated with TS, such as prefrontal, orbital, and cingulate cortices.

Alternatively, altered thalamic regions (especially the pulvinar nucleus) in TS patients may be due to hyperactive motor circuitry and compensatory mechanisms derived from years of attempting to control tics. In consideration of widespread thalamic afferents and efferent, increased thalamic grey matter volumes in TS patients may be associated with multisensory integration in the thalamus and/or convergence of sensory inputs to cognitive-, motivational-, and /or movement-related cortical areas [13]. It is noteworthy that CSTC circuit dysfunction is thought to cause the occurrence of tics [24]. The pulvinar nucleus is a prototypic association nucleus that takes part in reciprocal cortico-cortical interactions and accelerates synchronized oscillatory activities in functionally related areas of the cortex [22].

### Putamen

Findings from studies on the volume of the putamen in children with TS are inconsistent and have mostly focused on grey matter. For example, some studies have found smaller putamen in children and adults with TS [25, 26], while other studies have shown increased putamen volumes in such patients [13, 27, 28]. In our present meta-analysis, we found that the grey matter volume of the putamen was increased in TS patients. Interestingly, the dorsolateral putamina play a crucial role in motion planning by projecting the sensorimotor region [29–31]. The possible explanation for our finding is that the volume expansion of the dorsolateral putamina in TS patients might be caused by increased motor activity.

### Other regions associated with TS

In addition, we also found a decreased grey matter volume in the postcentral gyrus (motor-sensory gyrus), which indicates that primary motor pathways may be associated with TS [5]. It has been suggested that sensory-motor pathways may take part in the modulation of tics [32]. In contrast, grey matter volumes in insula have yielded mixed results. Some studies have found smaller insular cortices in children and adults with TS [24], whereas other studies have reported increased grey matter volumes in insular cortices of TS patients [5]. Regardless, these findings suggest that the insular cortex may play an important role in premonitory urges (PUs) [33, 34], which are based on interoceptive awareness [35]. Future studies are needed to further elucidate the role of the insular cortex in PUs.

Additionally, our present meta-analysis revealed decreased grey matter volumes in the medial frontal gyrus, which is consistent with several previous TS studies. Numerous studies have reported reductions in grey matter volumes in prefrontal cortices in TS patients, especially in the orbitofrontal cortex (OFC) [5, 13, 36]. For example, there is a negative correlation between OFC grey matter volume and tic severity [37]. Additionally, decreased grey matter volumes in the OFC have been reported in children with TS [13]. It was reported that cortical thickness of the right OFC decreased with age compared to the control group [38]. Besides, studies of adult TS patients showed a decrease in OFC grey matter volume, OFC cortical thinning [36, 39]. Changes in OFC activity may involve sensory aspects of twitching. These “uncomfortable feelings or bodily sensations” are referred to as PUs, which have a strong correlation with tic symptoms [40]. Task-specific fMRI studies should be performed in the future to explore the correlation between OFC activity and PUs in TS patients. Additionally, it seems that reduced OFC is found not only in children but also in adults with TS; although the volume of OFC found in this study was small, due to its important role in TS, future studies should follow up on this finding.

In our present meta-analysis, we found that grey matter volumes were reduced in the anterior cingulate gyrus (ACC) in TS patients. Previous studies showed cortical thinning and/or below-normal volumes in the subgenual ACC (sACC) [41]. The severity of the tic was also negatively correlated with tissue change in ACC on the right [37]. From functional imaging studies, tic production may be caused by changes in ACC [33, 42]. ACC activity increased with tic suppression, but ACC hypoperfusion was found in the quiescent period without tic suppression [42]. There was also a positive correlation between tic frequency and ACC activity [41, 43]. ACC abnormalities, however, might appear to play a key role in TS pathology.

In summary, with the development of neuroimaging, MRI technology includes voxel-based morphometry (VBM) technology, diffusion tensor imaging (DTI), resting-state fMRI to make the research on neurological dysfunction of TS is further in-depth. Although the research conclusions are not completely consistent, this may be due to different research groups, but MRI studies support the existence of structural and dysfunctional CSTC loops in TS patients, which provide a basis for further research in the future [44]. Overall, the brain regions with grey matter abnormalities in TS patients that we reported in our present meta-analysis were mainly located in the CSTC circle. Future studies employing other methodologies (e.g., functional imaging and neural networks) will be needed to determine the role of the CSTC circuit in TS.

### Limitations

Three limitations of our study should be acknowledged. First, the results reported in our meta-analysis comprised only six studies; future studies are needed with larger sample sizes. Second, several other factors (e.g., medications and comorbidities) should be considered in future MRI studies; we acknowledge that these factors may have accounted for the structural changes that we found in TS patients. Third, we only used the FDR to correct for our *p* values, while we did not use the family-wise error (FEW).

## Conclusion

In the present study, we primarily found that TS patients exhibited grey matter increases in the thalamus and lentiform nucleus, and grey matter decreases in the anterior cingulate gyrus. Most regions identified were associated with CSTC circuitry. Further studies with large sample sizes are needed to confirm these changes in grey matter volumes in TS patients.

## Supplementary Information

**Additional file 1.**

## Data Availability

The datasets used and/or analyzed during the current study are available from the corresponding author on reasonable request.

## Abbreviations

TS: Tourette syndrome
ALE: Activation Likelihood Estimation
MRI: Magnetic Resonance Imaging
CSTC: Cortico-Striato-Thalamo-Cortical
VBM: Voxel-Based Morphometry
MNI: Montreal Neurological Institute
YGTSS: Yale Global Tic Severity Scale
OFC: OrbitoFrontal Cortex
ACC: Anterior Cingulate gyrus
FDRs: False Discovery Rates
FEW: Family-Wise Error
PUs: Premonitory Urges
HC: Health Control
GMV: Grey Matter Volume
WMV: White Matter Volume
GMT: Grey Matter Thickness
PSP: Premonitory Sensory Phenomena
DOI: Duration Of Illness
BA: Brodmann area
L: left
R: right
