# Supplementary material for: Grey matter abnormalities in Tourette syndrome: an activation likelihood estimation meta-analysis
Source: BMC Psychiatry. 2021 Apr 7;21:184. doi: 10.1186/s12888-021-03187-1 (PMC8028086; doi:10.1186/s12888-021-03187-1)
Supplement: Supplementary file 1 — Additional file 1. [file 12888_2021_3187_MOESM1_ESM.docx]

| **Author** | **Year** | **YGTSS**  **x±s** | **N** | **Age**  **x±s** | **Males**  **%** | **DOI** | **Task** | **Main Findings** |
| --- | --- | --- | --- | --- | --- | --- | --- | --- |
| Clare M. Eddy | 2016 | 53.60±13.57 | 25 | 31.5±11.5 | 76.00 | 23.76±11.24 | Theory of Mind (ToM) | Neural activation was contrasted across ToM trials involving reasoning about false-belief, and matched trials requiring judgments about physical states rather than mental states. Contrasting task conditions uncovered differential fMRI activation in TS during ToM involving the right temporo-parietal junction (TPJ), right amygdala and posterior cingulate. Further analysis revealed that activity within the right TPJ as localized by this task co-varied with the severity of symptoms including echoing tics, impulse control problems and premonitory urges in TS. Amygdala activation was also linked to premonitory urges, while activity in the left TPJ during ToM was linked to ratings of non-obscene socially inappropriate symptoms. |
| Hongwei Wen | 2017 | 44.66 ± 17.96 | 29 | 8.76 ± 3.136 | 79.31 | 1.61 ± 1.38 | Topological disruptions | we use graph theory analysis to investigate the topological disruptions between groups. The identified disrupted regions in FC networks not only involved the sensorimotor association regions but also the visual, default-mode and language areas, all highly related to TS. |
| C. M. Eddy | 2016 | 53.60±13.57 | 25 | 31.48±11.50 | 76.00 | 23.76±11.24 | The standard version of the Reading the Mind in the Eyes Task | During prompted mental state recognition, greater activity was apparent in TS within left orbitofrontal cortex, posterior cingulate, right amygdala and right temporo-parietal junction (TPJ), while reduced activity was apparent in regions including left inferior parietal cortex. Age judgement elicited greater activity in TS within precuneus, medial pre- frontal and temporal regions involved in mentalizing. The interaction between group and task revealed differential activity in areas including right inferior frontal gyrus. Task-related activity in the TPJ covaried with global ratings of the urge to tic. |
| Gong-Jun Ji | 2016 | 20.29±6.12 | 24 | 9.46±2.19 | 100 | 2.29±1.60 | Amplitude of low-frequency fluctuation (ALFF) and functional connectivity | Amplitude of low-frequency fluctuation (ALFF) and functional connectivity were used to estimate the local activity in Globus pallidus internal (GPi) and its functional coordinate with the whole brain regions, respectively. We found decreased ALFF in patients’ bilateral GPi, which was also negatively correlated with clinical symptoms. Functional connectivity analysis indicated abnormal regions within motor and motor-control networks in patients (inferior part of sensorimotor area, cerebellum, prefrontal cortex, cingulate gyrus, caudate nucleus, and brain stem). Transcranial magnetic stimulation sites defined by previous studies (“hand knob” area, premotor area, and supplementary motor area) did not show significantly different functional connectivity with GPi between groups. |
| S. Bohlhalter | 2016 | 31±11.2 | 10 | 31±11.2 | 40 | 24±11.6 | Event-related functional MRI | On the basis of synchronized video/audio recordings, fMRI activities were analyzed 2 s before and at tic onset irrespective of the clinical phenomenology. We identified a brain network of paralimbic areas such as anterior cingulate and insular cortex, supplementary motor area (SMA) and parietal operculum (PO) predominantly activated before tic onset. In contrast, at the beginning of tic action, significant fMRI activities were found in sensorimotor areas including superior parietal lobule bilaterally and cerebellum. The results of this study indicate that paralimbic and sensory association areas are critically implicated in tic generation, similar to movements triggered internally by unpleasant sensations, as has been shown for pain or itching. |

**Abbreviations: YGTSS, Yale Global Tic Severity Scale; DOI, Duration of Illness.**

**sTable 1. Demographic characteristics of subjects and Summary of fMRI studies of TS**
